# An Artificial Intelligence-Based Virtual Human Avatar Application to Assess the Mental Health of Health Care Professionals: A Validation Study

**DOI:** 10.1089/jmxr.2024.0016

**Published:** 2024-10-18

**Authors:** Nicole R. DeTore, Oyenike Balogun-Mwangi, Elizabeth S. Eberlin, Katherine N. Dokholyan, Albert Rizzo, Daphne J. Holt

**Affiliations:** 1 Department of Psychiatry, Massachusetts General Hospital, Boston, Massachusetts, USA.; 2 Department of Psychiatry, Harvard Medical School, Boston, Massachusetts, USA.; 3 Department of Psychology, Bentley University, Waltham, Massachusetts, USA.; 4 Institute for Creative Technologies, University of Southern California, Los Angeles, California, USA.

**Keywords:** mental health assessment, health care workers, human virtual avatar, artificial intelligence, mobile application

## Abstract

Many health care workers experience high levels of stress, mental health issues, and burnout, yet are less likely than most to seek mental health support. Given this challenge, non-stigmatizing approaches that promote access to assessments of mental health for this population are greatly needed to increase self-awareness and connection to care. Previous research has shown that when users engaged in dialog with a virtual human agent (VHA), they disclosed more information about their mental health compared with similar interactions with a live human or human-as-avatar assessor. An application called “BeCalm” was developed to conduct psychological assessments using conversational artificial intelligence and user interaction with a human-like VHA. BeCalm allows for both spoken and written “chat”-based communication. This pilot study aimed to measure the user experience, acceptability, and convergent validity of BeCalm. A cross-sectional, mixed-methods, one-arm study of BeCalm was conducted with 38 health care workers (mean age = 31.87, standard deviation = 11.28; 84% biologically female). Qualitative interviews indicated that the simulated interpersonal connection with the VHA was the most appealing aspect of BeCalm, with participants describing the VHA as warm and nonjudgmental. The second most highly rated aspect of the application was the information (resources, summary, and psychoeducation) provided at the end of the assessment. There was a range in levels of convergent validity between the BeCalm and conventional assessments across several symptom domains (*r*s = 0.101–0.766), with mood and occupational burnout assessed with the highest validity. Notably, spoken verbal interactions with the VHA elicited longer participant responses by an average of 60 characters compared with chat interactions. In summary, BeCalm can provide a valid mental health assessment and resources for an at-risk population through interactive technology and personalized feedback. BeCalm offers a user-friendly, scalable method for assessing health care workers’ mental health that could lead to behavioral change.

## Introduction 

Health care professionals commonly experience emotional burnout, distress, and higher rates of mental illnesses compared with the general population.^1–4^ Suicide rates among medical providers exceed those of the general population by almost twofold and have increased further since the pandemic.^5–8^ Although numerous treatment options are available, health care professionals often report stigma-related barriers to seeking treatment, which impact their ability to receive appropriate assessment and care.^9,10^ Concerns about being perceived as weak or unable to handle professional responsibilities deter them from seeking help or taking time off for mental health treatment,^11^ and are even observed in health care providers specializing in providing mental health services.^12^ Worries about confidentiality and the potential impact of receiving mental health treatment on career advancement, including fears of losing licensure or job opportunities, are also common.^13,14^ More than 40% of physicians report that they would be reluctant to engage in mental health treatment due to these concerns.^15^ In addition, the long working hours and high job stress associated with health care careers not only contribute to mental health issues but also leave little time to obtain mental health care if needed.^3^ Therefore, despite their knowledge of health and access to resources, health care workers’ willingness and ability to engage in mental health treatment are significantly impacted by these barriers.

One novel approach for addressing some of these barriers is to provide mental health assessments and information using a virtual assessment tool that can be highly confidential and flexibly used. Virtual assessment tools can allow busy providers to log in when they want, where they want, and for as long as they would like. There is also evidence that speaking with a virtual human agent (VHA) or avatar, rather than a real human, about mental health symptoms is preferable for many people, mitigating concerns regarding stigma.^16^ Thus, deploying VHAs that interact with users via natural language processing, and conversational artificial intelligence (AI) technology for mental health assessments, may improve access to mental health treatment, as they can be engaging, scalable, and confidential.^17,18^ They also can be tailored to the interests and needs of the user and accessed at the user’s own pace when convenient.^19,20^

Previous studies found that users disclosed more personal information, experiences of sadness, and symptoms of psychopathology when interacting with a VHA, reportedly due to having less concern about being judged, compared with interacting with a real person.^18,21^ The digital assessment programs that are available currently utilize various modalities such as virtual reality, ecological momentary assessment, and passive mobile-based markers.^22,23^ However, few have been fully validated before becoming available to the public.^5^ Chatbot-style applications have been developed to deliver mental health interventions, with some focused on screening for a specific disorder. While the development of applications using AI-based VHAs for mental health assessment has begun, none specific to health care providers have yet to be tested.^24,25^

In light of this gap, we developed an application called “BeCalm” to conduct psychological assessments using conversational AI and user interaction with a digital, human-like VHA, covering a wide range of topics from mood and anxiety to occupational burnout. The application also provides self-help education, online resources, and information regarding resources for obtaining professional support or clinical care if needed.

Thus, the primary aim of the current study was to evaluate the user experience, acceptability, and convergent validity of the BeCalm application among health care workers. We tested whether BeCalm is feasible, acceptable, and has convergent validity with standard, well-established measures of symptoms of psychopathology.

## Method

### Overall design

This mixed-methods pilot study of the BeCalm application examined the user acceptance and construct validity of intra-app user measurements. This study solicited user feedback, examined application completion rates, and measured the application’s convergent validity compared with both self-report and clinician-rated assessments.

### Recruitment

Study recruitment was conducted through a posting on a website dedicated to recruitment for research studies conducted in the Mass General Brigham (MGB) health care system (rally.massgeneralbrigham.org). Eligibility criteria included: (1) employment within a health care system, regardless of the amount of patient contact, (2) age 18 or above, and (3) fluent in English, both written and spoken. These criteria were assessed through a brief phone call and receipt of an email from the participant with the email address of the medical setting where they were employed. All participants provided written informed consent prior to participating, and all procedures of this study were approved by the MGB Institutional Review Board.

A total of 38 participants, who were currently employed in various roles at a hospital, completed this study. The average age of the participants was 31.87 (standard deviation [SD] = 11.28) and 84% were biologically female. See Table 1 for the demographic characteristics of the participants.

**Table 1.** Participant Demographic Characteristics (*n* = 38)

Characteristic	M	SD
Age	31.87	11.28
Years of education	16.95	1.74

	***N***	**%**
Sex		
Male	5	13.2
Female	32	84.2
Trans male	1	2.6
Marital status		
Never married	28	73.7
Married	9	23.7
Divorce	1	2.6
Ethnicity		
Hispanic	4	10.5
Not Hispanic	34	89.5
Race		
Asian	9	23.7
Black or African American	1	2.6
White	29	73.7
English as a first language	33	86.8
Health care role		
Clinical research	10	26.3
Physician	6	15.8
Technician	6	15.8
Nurse	6	15.8
Mental health clinician	6	15.8
Hospital administrator	4	10.5
Born in the United States	5	13.2

M, mean; SD, standard deviation; N sample size; % Percentage.

### The BeCalm application

#### Application development

**FIG. 1.**
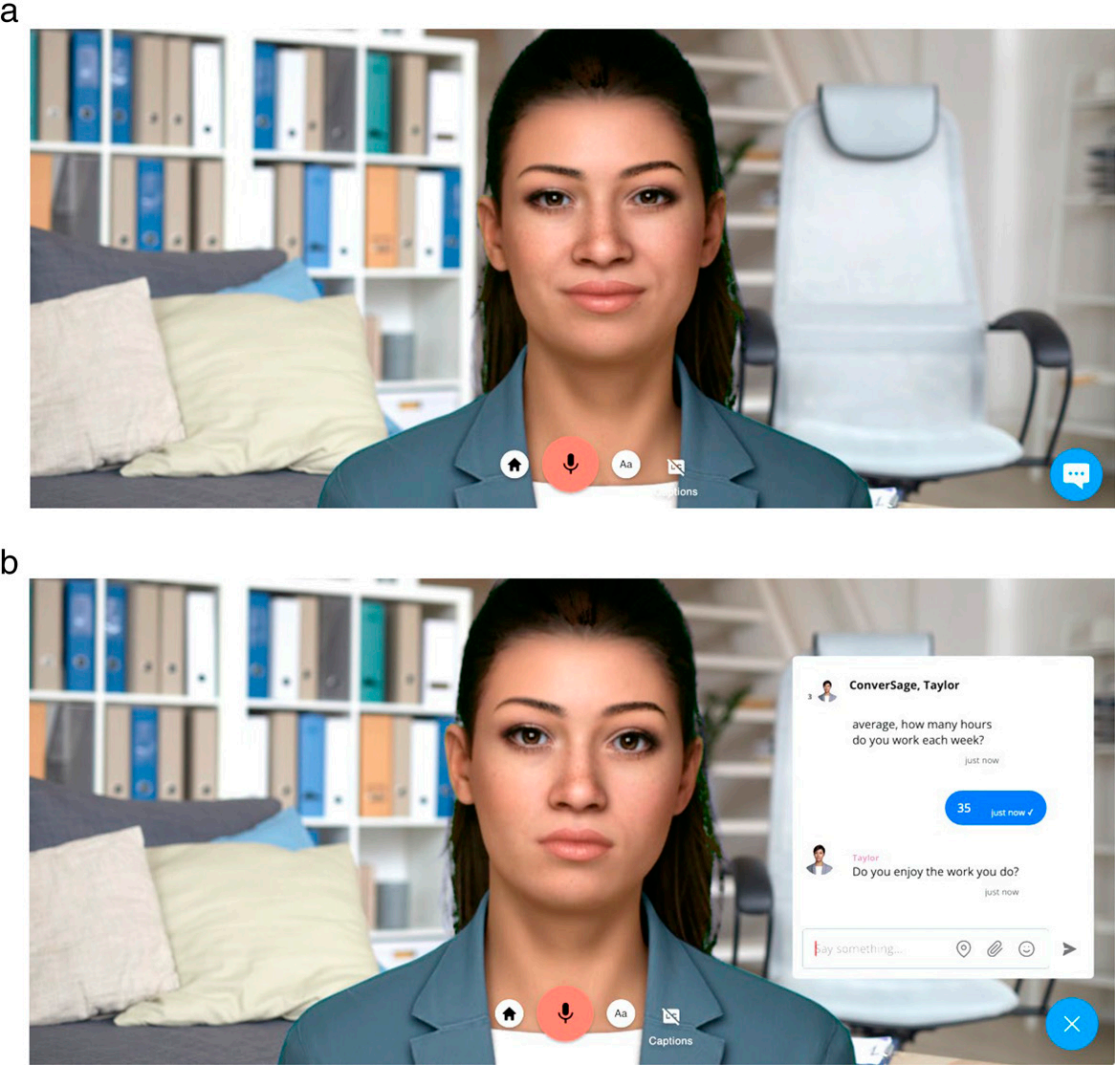
Examples of the forms of communication with the BeCalm virtual human avatar: **(a)** voice and **(b)** chat.

The BeCalm application was built utilizing outsourced, cloud-based services in collaboration with ConverSage, a health care training company, and a private technology company, eXtended Intelligence. The VHA was programmed to understand the basic intent and context of questions. The application provides the dialog framework to utilize a question-and-answer dialog approach, using a conversational interface based on natural language processing, implemented with speech-to-text and text-to-speech tools. BeCalm can interact with users via either spoken (the user speaks aloud) or written (the user types in a chat box) communication, with the option to change this setting (switch to the other option) at any time throughout the use of the application. Users can also control how the VHA communicates with them, either via speaking or through text responses in the chat box, which can also be adjusted throughout the use of the application. Examples of both forms of communication are shown in Figure 1a and b.

After the 27th participant was enrolled, it became clear from participant feedback that the coding accuracy of the spoken responses of participants was inadequate, with 80% of participants noting that the VHA was unable to understand their spoken responses and six participants referring to this as “annoying” or “frustrating.” Therefore, between subjects 27 and 28, the AI large language model ChatGPT 4.0 was integrated into the application to improve its speech processing accuracy. We found no evidence of any impact of this change on symptom validation, only on the user experience (see BeCalm usability and acceptability results section).

#### Application description

Participants completed the BeCalm mental health assessment via interactions with a VHA named “Taylor” (see Fig. 1). The BeCalm application was sent to participants via a link in an email and could be used on a computer, tablet, or mobile phone.

The VHA begins the assessment by briefly introducing the participant to the application, what to expect, how to use it, detailing how they can end the assessment, or pause it, as well as describing the confidentiality of the assessment. The VHA then asks questions about the demographic characteristics (e.g., age and sex) of the participant, and then asks questions related to each of the nine mental health domains covered in the assessment. The VHA was programmed to also present follow-up statements of empathy, interim summaries, and normalization as appropriate, using a complex conversational interface with content, logic, and scoring.

Motivational interviewing (MI) was chosen as the theoretical model for BeCalm, with the goal of building motivation for change, due to the efficacy of MI in supporting at-risk populations in engaging in mental health treatment,^26,27^ as well as the reported efficacy of MI for boosting engagement with chatbot-style applications.^28^ This involved the use of open-ended questions, affirmations, reflections, and summaries.

#### Application assessment and content

All language content, including the BeCalm assessment questions, the scoring for each question, logic and algorithm design, follow-up responses, and answers to common questions, was generated by two PhD-level clinical psychologists. The mental health domains assessed by the application include occupational burnout, professional quality of life, general quality of life, sleep, anxiety, loneliness, substance use, psychosis, and mood. These domains were chosen based on previous research demonstrating that these are domains that are affected in health care workers.^1,2^ Each mental health domain included 3–17 questions (each developed specifically for this application), and each domain began with 2–3 initial questions to determine if the user should continue to receive additional questions in that domain or move to the next. This means that users who did not report any or few mental health issues completed a shorter version of the assessment (lasting ∼11 min) than those who reported symptoms. This was done to ensure that participants would not have to answer more than three questions on topics that were not specifically related to their own mental health needs.

Responses to each question were given a three-level threshold: positive (2), neutral (1), or negative (0). A vast array of possible responses was then categorized into one of those three levels by the two clinical psychologists who designed the questions not only to determine the answer scores but also whether an empathetic, summarizing, or normalizing response should be given. A previously unvalidated algorithm was designed to determine which question would be asked next, depending on the response to the prior question. From there, the score for the answer to each question within a domain would be calculated to determine a domain total score, which had a 4-level (not present, mild, moderate, and severe) range.

Once the users completed the assessment portion of the BeCalm application, they were presented with a brief 5-line summary describing each area of concern for that user (any domain rated mild or above), and an indicator of the severity level of their self-reported ratings using the same 4-level range. This summary included psychoeducation for each area of concern, including relevant links to additional information and resources for obtaining support for each area of concern using MI techniques.^29^ To validate this rating process, we conducted a convergent validity analysis comparing the BeCalm assessment with previously validated evidence-based assessments.

### Evidence-based measures for convergent validity comparison

#### Self-report measures

Within one day of completing BeCalm, participants were emailed a survey link that directed them to a battery of self-report screening measures. This survey included evidence-based self-report assessments selected to measure the same domains that were assessed in the BeCalm application.

To assess occupational burnout and quality of life, two scales were used: (1) the Professional Quality of Life Scale,^30^ a 30-item self-report questionnaire comprised of three discrete subscales measuring compassion satisfaction, burnout, and compassion fatigue/secondary trauma, and (2) the Maslach Burnout Inventory,^31^ a 22-item assessment that measures burnout with three primary scales: emotional exhaustion, depersonalization, and personal accomplishment (and a total score). To assess sleep, the brief 7-item Insomnia Severity Index,^32^ which assesses the severity of nighttime and daytime elements of insomnia, was used. To measure mood, the Patient Health Questionnaire-9,^33^ measuring the severity of depression and symptoms of anhedonia, and the Beck Depression Inventory,^34^ a 21-item assessing symptoms of depression including depressed mood, pessimism, and social withdrawal, were used. Finally, the UCLA Loneliness Scale,^35^ a 20-item measure, was used to capture the experience of loneliness and social isolation.

#### Clinical interview measures

The Mini International Neuropsychiatric Interview (MINI)^36^ was conducted within a week of the participant completing the self-report surveys. The MINI is a well-validated clinical interview that uses Diagnostic and Statsitical Manual of Mental Disorders-5 (DSM-5) diagnostic criteria to determine the presence of psychiatric diagnoses across multiple domains including mood, anxiety, substance use, psychotic, and eating disorders.

### User feedback measures

#### Quantitative assessment

Following the completion of BeCalm and the assessments, participants completed a self-report feedback form. This form obtained ratings on a 5-level Likert scale (strongly disagree, disagree, neutral, agree, strongly agree) that assessed elements of usability, preferences, and impact of BeCalm, evaluating whether they learned about reasons to embrace personal change, felt they would change their behavior based on what they learned from the application, and whether they would recommend BeCalm to a colleague.

#### Qualitative interview

Participants then completed a 5–15-min Zoom-based qualitative interview with study staff, which obtained feedback on BeCalm using an 8-question, semistructured, open-ended interview guide (see Supplementary Data S1 for the qualitative interview guide). Participants were asked what they liked and disliked about the experience, how the BeCalm application compared with other surveys the participant had completed in the past, what they found therapeutic about BeCalm if anything, what they found unhelpful, and whether they had any other feedback about the application that they would like to share (positive, negative, or otherwise). The interview ended once the participant reported that they had shared all relevant thoughts about their experience with BeCalm.

### Statistical analysis

To assess the usability and acceptability of BeCalm, we calculated the application’s completion rate and the amount of time participants spent using the application. In addition, we measured frequencies and percentages of the acceptability ratings from the quantitative feedback forms. Frequencies of communication with the VHA via verbal speaking versus the chat feature were also compared, and paired *t*-tests were used to calculate differences between the average length of responses for each feature. Pearson correlations were used to examine relationships between the length of responses, the number of responses for the nine BeCalm domains, and the quantitative feedback ratings. Partial correlations were used to control for the type of interaction (spoken verbally or through the chat feature) for any significant relationships found.

Qualitative interviews were audio recorded and then transcribed verbatim using NVivo software. Transcriptions were then independently reviewed and coded for themes using a grounded theory approach^37,38^ by two independent coders (one PhD-level psychologist and one master’s-level researcher) trained in analyzing qualitative data. All transcripts were then reviewed by the two coders to determine consensus and to identify the main themes. Frequencies of endorsement of each theme were then calculated.

To determine convergent validity, Pearson correlations were calculated to compare the BeCalm severity ratings and the scores on the self-report survey subscales, using total scores for each corresponding domain. Similar Pearson correlations were also computed to compare the BeCalm domain ratings and the MINI domain scores. Finally, chi-square analyses were used to determine whether the type of communication preferred with the VHA was related to any BeCalm symptom domains or overall severity of ratings.

## Results

### BeCalm usability and acceptability

Of the 38 participants, 100% completed the full BeCalm assessment. Participants spent an average of 22.25 min using the application (SD = 7.52), with a maximum of 39.52 min and a minimum of 11.47 min. The majority of participants (63.2%) logged into the application more than one time, with an average of 2.61 (SD = 3.77) times, to complete the assessment. The remaining participants (36.8%) completed the assessment in one sitting. Across all participants, the average number of responses was 69.87 (SD = 17.99), and the average length of response in characters was 12.43 (SD = 9.40).

**FIG. 2.**
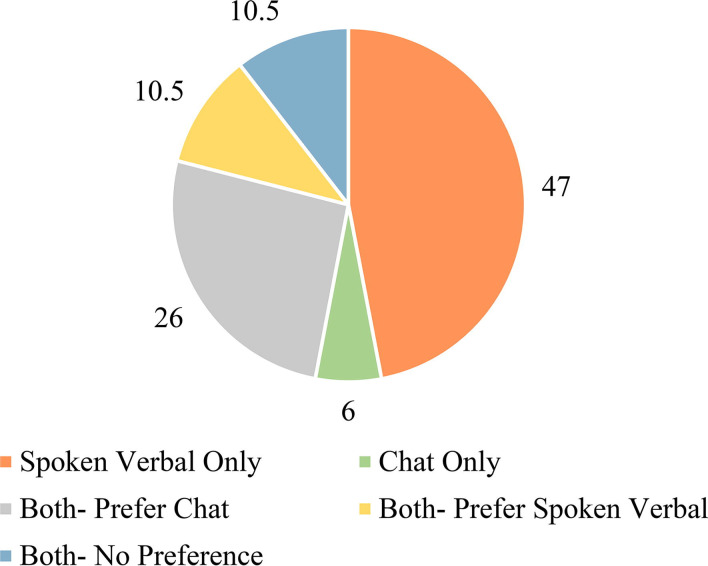
BeCalm user preferences for the form of interaction with virtual human avatar.

There was an approximately equal split between those participants who communicated with the VHA only using the spoken verbal option (47%; *n* = 18) and those who used both the speaking and chat options (47%; *n* = 18). Only 5% (*n* = 2) of participants exclusively used the chat feature. Of those who used both options, 78% (*n* = 14) of participants used only one feature for 10% or less of their interaction before switching to the other. Most of those participants (71%) made the switch from the spoken to the chat option, and the remaining only briefly used the chat feature. In sum, 39% of the total sample used the chat feature for the majority of their communication (see Fig. 2). Following the incorporation of ChatGPT, there was a significant increase in both the length of response (*t* = 2.15, *p* = 0.037) and the proportion of users who used the spoken verbal option (*X*^2^ = 20.08, *p* < 0.001), suggesting that the early users may have used the chat option due to BeCalm’s initial, more limited ability to decode spoken language.

In addition, there was a significant difference in the length of the interaction between the spoken conversations and the chat conversations (*t* = −4.52, *p* < 0.001), with a mean length of 70.5 characters and a median of 52.4 for the spoken responses, and a mean of 10.3 characters and a median of 6.2 for the chat responses.

A majority of the sample (53%; *n* = 20) reported that they would use the BeCalm application again if it was available to them, whereas 15 said they would not, with 8 of those 15 indicating that they did not need it because they were already seeing a therapist. Six participants reported that they were not currently experiencing any mental health-related distress but would recommend it to others. Finally, one person reported that they did not enjoy the application and the remaining three participants did not answer that question.

A total of 47.3% (*n* = 18) of participants endorsed (rated that they agreed or strongly agreed with) the statement that they “learned a reason to embrace personal change” through the BeCalm application, and 42.1% (*n* = 16) felt that they would change behavior based on what they learned from the application. Also, 42.1% (*n* = 16) reported that BeCalm would be useful to a colleague.

### Qualitative data

Qualitative interviews with all 38 participants revealed that the most appreciated feature of BeCalm was the interpersonal connection that they felt they experienced with the VHA, which was perceived as warm, approachable, and nonjudgmental. The resources, summaries, and psychoeducational materials provided after the assessments were the second most valued aspects, offering new, actionable knowledge that participants felt was not readily available through simple online searches (i.e., “not something I could just google”). The third most valued feature was the assessment content itself; participants appreciated the personalized, detailed questions specifically tailored to health care professionals. In addition, participants highlighted the application’s user-friendly interface and accessibility, noting its convenience for use anytime, anywhere, and the option to switch between speaking and typing. Many (*n* = 29) found the application to be therapeutic, especially for the insights it offered into their mental health through summaries, psychoeducation, and resources. Several participants (*n* = 5) also found the act of speaking to the VHA to be therapeutic. However, the primary criticism (reported by 80% of the first 27 participants) was the VHA’s occasional misunderstanding or misinterpretation of spoken responses, which some found frustrating and chose to circumvent by typing instead. Participants described this as poor comprehension (by the VHA) of their verbal responses. In response to this feedback, we incorporated ChatGPT 4.0 into BeCalm to assist with decoding verbal responses for the remaining 10 participants. This addition led to improved ratings, with a 60% decrease in participant reports of comprehension errors. Participant themes and representative quotes from the qualitative interviews are provided in Table 2.

**Table 2.** Participant Quotes from Qualitative Interview (*n* = 38)

Themes	*n*	Participant Quotes
Positive attributes of the BeCalm application		
Interpersonal connection with the VHA (“Taylor”)	18	“Taylor is like my best friend who I talk about everything, from bad to worse, yeah. She’s not going to judge me; she’s going to listen to me. She’s going to give me some recommendations, and always she is smiling and doesn’t make me feel discriminated. Yeah”—02 “Even though it was just a graphic on the screen, it wasn’t a real person, I think there was some sort of almost human connection that you could kind of feel and you felt like you were having a conversation with somebody, and it offered some helpful resources too. Like, I found the feedback at the end was quite helpful, some of the resources at the end. So, I found that helpful and I think in general I felt it was a good resource just because it’s it can be hard to find like real in-person therapy and things like that. So, it was a nice kind of way to get some things off your chest that might be hard to discuss with an actual live person, therapist, you know, whatever you want to call it. It’s nice to have sort of something that’s well trained in some sort of psychology, but not have to worry about that judging you.”—16
Resources, summary, and psychoeducation	17	“I thought it was cool that it recommended resources based on your answers. So, you know, if you said that you were having troubles. I said that I had a little bit of trouble sleeping and it recommended some different sleep hygiene techniques. I thought that that was cool, that your concerns were kind of answered with something that you could use to address them.”—01 “The report that it generated, I thought it was eye opening because I knew there were some problems. Yeah, but I wasn’t like self-aware enough to admit some of them. It was like, ‘You’re really high risk of this,’ and I was like, ‘Damn, I guess you’re right.”—18
Assessment questions	16	“I really like the progressive questioning of it that I kind of built up the conversation to kind of, I guess, get into the specifics of what your experience was working in health care.”—27 “Taylor is asking many questions and all of that information, and the algorithm is going to mix the information and cook them together and give you a cake that suggests how to move forward and what to do. It is something personalized; it is not just like an Alexa that gives you pure facts and information. I have always been interested in going to a psychologist, but I believe that psychologists have mental problems so they cannot help me. Taylor is like a psychologist that doesn’t have psychologist problems.”—02
User friendly and accessible	15	“That there are like multiple ways to communicate with Taylor. So, like, if I mean, I think I mainly use the chat tool and I didn’t really use the microphone very much, but, you know, maybe someone is using this screening in another area where they don’t have their laptop or don’t have their hands available. So having like both of those options I thought was cool that it was available.—27 “I do like the accessibility of it. I think a lot of people use their smartphone and just having to not have to go into an office and do stuff, that’s a lot easier. And maybe it feels a little bit less intimidating that it’s not an actual person in some ways.”—15
Qualities of the VHA (positive)	8	“I think, like, it [the VHA] looked friendly and it looked like if you were just like in therapy or something and speaking with someone and she was giving you some resources that you could use to just like help yourself or she was helping you. So, I think like the interface, like the face that you guys gave her and everything, it looked friendly and approachable and the voice too. It was like, calming.”—23
Negative attributes of the BeCalm application		
Challenges understanding spoken responses	28	“One of the biggest issues I had is that it didn’t understand a lot of my responses that I was giving, like verbally. And they were things that I thought were pretty standard responses to the questions.”—22 “But then sometimes she didn’t understand my responses. And so, it just like took more time and was kind of more frustrating to get across.”—08
Qualities of the VHA (negative)	7	“I think her voice is kind of stiff. It’s a little rigid and it’s like the tone does not change, and sometimes it just feels a little monotonous.”—32
Virtual aspect	7	“I guess like the AI aspect, I found like it would have even been more helpful if it were a real person.”—19 “Yeah, it just I mean, it obviously was artificial, but it felt artificial. And like, I think what I can read pretty quickly and so it just like becomes easier to, to read it. But I also just like how like, who is this AI person pretending to know things about me? So yeah, it just didn’t feel real, which obviously it wasn’t.”—30
Concerns about AI	4	“And, you know, there was a bit of a like an uncanny valley, kind of like, you know, sometimes it was just weird watching her facial expressions. And sometimes I would kind of forget that she was AI. And other times I would feel kind of uncomfortable with it.”—35
Therapeutic aspects of the BeCalm application		
Questions and conversation provided new insights	14	“Just because it kind of helps you keep track of yourself. I’d like to think that I’m pretty self-aware, but obviously I wasn’t. And this can kind of keep track of the symptoms.”—18 “It made me think more deeply about how my work experience was for me when I was when I was losing it. I was like, you know, in a rut with work and I was feeling pretty frustrated and burnt out. And I think using the BeCalm app helped me realize that and also made me think a little bit more about how some of the questions that I might not have associated with burnout and like other work-related stressors affected, were affected by burnout.”—29
Nothing	9	“Honestly, I think that she kind of just told me what I really knew at the end, with the anxiety/ depression and so I didn’t really find it very helpful or anything.”—20
Resources, summary, and psychoeducation	8	“I would say just the fact that it gets you thinking about your experience working in the health care system, especially in a time where I kind of just wanted to not think about it when I got home, it kind of forced me to, which I think was therapeutic in a way. And I think if I had received the resources that would have been helpful to look at that too. Because if I remember correctly, at the end it says like, ‘oh, here’s where we see you’re having issues,’ and then you scroll to the next part and it’s like, ‘here’s what might help you.’ Yeah, so even just having it was almost as validating in a way, for the program to tell me what they saw was going on from my responses. Yeah, and then to be provided resources, also helpful.”—29
The BeCalm application compared with web-based surveys		
BeCalm was more engaging	19	“I thought it was a lot better than most surveys were. Mostly just like yes or no-click through. Well, this one was felt very personalized.”—17
Prefer web-based surveys	6	“I personally prefer, like the kind of, like self-paced, like looking at online training questions. I thought I was it felt not much more personal just because it was on a computer screen. I think like, a click off for me would have done probably the same thing.”—05
Similar to web-based surveys	6	“I think they’re about comparable. I don’t think it was any better or worse.”—17

AI, artificial intelligence; VHA, virtual human agent.

### BeCalm validity

Convergent validity between BeCalm responses and the self-report measures showed a large range across the symptom domains, with occupational burnout and mood showing the strongest validity (*r =* 0.317–0.766, all *p* < 0.065) and workplace satisfaction and substance use with the weakest. Convergent validity between assessments of the interview-rated symptoms obtained from the MINI and BeCalm responses also varied, with mood and psychotic experiences showing the strongest (*r* = 0.307–0.757, all *p* < 0.061) and substance use and panic disorder showing the weakest. MINI diagnoses of substance use disorders, panic disorder, and psychotic disorders were rare in this sample, which may explain the lack of significant convergence in two of those three areas. When the number of responses before and after the incorporation of ChatGPT was compared, there was no significant difference (*t* = 0.581, *p* = 0.568). The statistical results of these analyses are presented in Table 3.

**Table 3.** Correlations Between BeCalm Symptom Domains and Standardized Self-Report and Interview-Rated Symptom Domains

BeCalm Ratings	Self-Report Measures			Interview-Rated MINI		
	**Scale**	***r***	***p***	**Domains**	***r***	***p***
Anxiety	PHQ-9	0.454	0.004	Generalized anxiety	0.335	0.040
				Panic	−0.053	0.837
Mood	BDI	0.676	<0.001	Major depression	0.410	0.011
	PHQ-9	0.690	<0.001	Suicidality	0.388	0.016
	UCLA	0.449	0.005	Mania	0.307	0.061
Sleep	ISI	0.572	<0.001	—		
Substance use	—			Alcohol	0.101	0.547
				Substances	0.252	0.127
Loneliness	UCLA	0.408	0.011	Major depression	0.367	0.023
Psychotic experiences	—			Psychosis	0.757	<0.001
Burnout	MBI total	0.661	<0.001	—		
	ProQual	0.766	<0.001			
	Compassion	−0.526	<0.001			
Work-related trauma	ProQual work trauma	0.686	<0.001	—		
Quality of life	ProQual	0.431	0.007	—		
	Compassion	−0.350	0.031			
Satisfaction with work	MBI total	0.302	0.065	—		
	ProQual	0.317	0.053			
	Compassion	−0.390	0.015			

BDI, The Beck Depression Inventory; compassion, subscale of the Professional Quality of Life Scale; ISI, Insomnia Severity Index; MBI, The Maslach Burnout Inventory; MINI, The Mini International Neuropsychiatric Interview; PHQ-9, The Patient Health Questionnaire-9; ProQual, the Professional Quality of Life Scale; UCLA, the UCLA Loneliness Scale.

In addition, when associations between length of responses and severity of symptoms were assessed, we found that participants rating higher on loneliness used more characters to answer each question on average (*r* = 0.399, *p* = 0.013). This relationship remained significant even after controlling for the way in which the participants interacted with the VHA (*r* = 0.400, *p* = 0.014). No other symptom was linked with the length of participant responses (all *p*s > 0.073). Finally, the type of communication chosen by the participant (spoken verbal, chat, or both) was not significantly related to any BeCalm domains or the overall symptom severity reported by participants (all *p*s > 0.152).

## Discussion

### Principle results

This pilot user study measured user perceptions and the assessment validity of the BeCalm application, an innovative tool designed to assess and support the mental health of health care professionals via interactions with a VHA. With 76% of users reporting that BeCalm was therapeutic and 42% indicating that they would change some aspect of their behavior based on what they learned from the application, our findings suggest that the application may be a promising avenue for enhancing mental health knowledge and well-being in this population. Overall, health care providers described a positive user experience with BeCalm, often reporting that it provided insight and led them to want to change their behavior. It also showed convergent validity with previously validated mental health assessments in several symptom domains (e.g., burnout, mood), indicating that the feedback generated by the application was appropriately tailored to the specific experiences and symptoms endorsed by the participants. Moreover, the application’s resources, general summaries, and psychoeducation components were reported to be of value for their accessibility and relevance, offering useful, actionable information.

Users interacted with the application using a variety of conversational modes. The majority of users preferred spoken verbal interactions alone or mixed spoken verbal/chat interactions (57.5% used spoken verbal and 10.5% used both options equally). Those speaking with the VHA had significantly longer responses than those typing their responses, with an average of 60 more characters. Interestingly, those with higher loneliness ratings in BeCalm responded with longer answers across all domains, suggesting that a sense of connection may have been sought out (and potentially experienced) by this subset of users when responding to the VHA.

Feeling a connection with the VHA was the most frequently endorsed valued aspect of BeCalm. Moreover, several participants reported that speaking with the VHA was in itself therapeutic, in addition to the benefit of receiving the results of the assessment. This feedback is in line with previous literature showing that conversing with VHAs may provide psychological support for some users.^39^ Also, the summary at the end of the application that provided users with psychoeducation and local resources was the second most liked aspect of the application (45%).

### Limitations

The findings of this study must be interpreted with its limitations in mind. First, the sample size was modest, with just 38 participants. Second, the sample was predominantly comprised of White females. Thus, this study did not assess the experience with BeCalm in the wide range of individuals employed in health care professions with respect to race, ethnicity, and gender, as well as socioeconomic status. Future studies of BeCalm can examine its use in a more broadly representative sample and assess whether users would prefer to interact with a VHA whose appearance more closely represents their gender and ethnicity. Future updates of BeCalm can include a choice of VHAs that reflect the diverse identities of health care professionals.

Third, the validation of the BeCalm assessment was limited by the partial reliance on self-reported information about the participants’ symptoms of psychopathology, which can be biased and lead to underreporting of symptom severity.^40^

Lastly, BeCalm was designed to assess the mental health needs of individuals employed in a wide range of roles in health care, from clinicians to food service providers. The challenges and needs associated with these different roles are varied, yet experiences and symptoms such as burnout, loneliness, anxiety, and depression are observed across individuals employed in many professions, including those within the health care sector. Thus, the current results suggest that BeCalm can function as an easy-to-use tool for self-assessment and support of the mental health needs of this broadly defined population. Future iterations of BeCalm can also provide targeted feedback regarding specific concerns and challenges experienced by clinical versus nonclinical health care professionals and other subgroups of this heterogeneous category of employees.

It is also important to note that the initial iteration of the BeCalm application did not include voice processing technology that was sufficiently proficient in decoding the verbal responses of participants. This was addressed with the addition of ChatGPT 4.0 in an updated version. This adaptation was implemented and tested for the last 10 participants enrolled in the study and was well-received by participants. Thus, further refinement and testing of the application’s AI capabilities are needed to ensure that there is effective communication with users.

## Conclusions

The BeCalm application represents a novel approach to supporting the mental health of health care professionals. Its user-friendly interface and customized content are useful, appealing aspects of a new tool for assessing mental health in a profession known for highstress levels and experiencing many barriers to engaging in mental health assessment and treatment. The results of this pilot study demonstrate the application’s strong usability and acceptability, and that it is a valid mental health assessment tool across a wide range of domains. Thus, BeCalm can provide a confidential and effective pathway to care for a population that is often struggling with significant burnout, emotional distress, and a reluctance to seek professional help. Future research can also examine the utility of BeCalm for other populations in need of mental health assessment and support. Thus, BeCalm could contribute to closing the gap between the overall need for mental health assessment and access to treatment and the availability of appealing and effective solutions for this widespread societal problem.

## Abbreviations Used

%: percentage
AI: artificial intelligence
BDI: The Beck Depression Inventory
DSM-5: Diagnostic Statistical Manual-5
ISI: Insomnia Severity Index
M: mean
MBI: The Maslach Burnout Inventory
MGB: Mass General Brigham
MI: motivational interviewing
MINI: Mini International Neuropsychiatric Interview
N: sample size
PHQ-9: The Patient Health Questionnaire-9
ProQual: Professional Quality of Life Scale
SD: standard deviation
UCLA: UCLA Loneliness Scale
VHA: virtual human agent
